# Case Report: A review of two children with deep sternal wound infections after precordial surgery treated with a simple negative pressure closed drainage technique

**DOI:** 10.3389/fped.2024.1491944

**Published:** 2024-12-11

**Authors:** Yao Jiang, Shan He

**Affiliations:** ^1^ Department of PICU (or Pediatric Research Institute), Children’s Hospital of Chongqing Medical University, Chongqing, China; ^2^ Ministry of Education Key Laboratory of Child Development and Disorders, National Clinical Research Center for Child Health and Disorders, China International Science and Technology Cooperation Base of Child Development and Critical Disorders, Chongqing Key Laboratory of Pediatrics, Chongqing, China

**Keywords:** negative pressure, pediatric, congenital heart, deep sternal wound infection, treatment

## Abstract

Deep sternal wound infection (DSWI) is a rare but potentially devastating complication of median sternotomy performed in cardiac surgery. This report summarizes the nursing management of two pediatric cases with a DSWI treated using Do It Yourself (DIY) negative pressure suction (DIY-NPS) after surgery. The technique maintains a continuous suction pressure of 75 mmHg and intermittently flushes small volumes of fluid to stimulate granulation tissue formation and control systemic infection. After the formation of fresh granulation tissue, both patients achieved successful wound healing and were discharged in good condition.

## 1 Introduction

Approximately one-third of children with congenital heart disease have severe or complex cases, with the incidence in China estimated to range between 0.4% and 1.2% (1). To prevent hemodynamic instability caused by postoperative myocardial edema, approximately 10% of children undergo delayed sternal closure, typically performed 3–7 days after surgery (2). The incidence of thoracic incision infections following delayed closure is 8.5%–13.7%, with a secondary bloodstream infection rate of 6.9% (3, 4). Mediastinitis and deep wound dehiscence, collectively referred to as deep sternal wound infections (DSWIs), are key features of incisional infections. Studies conducted nationally and internationally report a DSWI prevalence of 1%–18%, with a higher incidence observed in younger patients. Furthermore, some studies indicate that the mortality rate associated with a DSWI ranges from 5.8% to 47% (2, 4–6). When a DSWI occurs, it significantly increases treatment costs, complicates management, prolongs hospital stays, and delays recovery. This imposes substantial burdens on families and the healthcare system.

Although closed continuous negative pressure suction is a safe and effective wound treatment, its application in pediatric patients with a DSWI remains uncommon due to the lack of standardized pressure guidelines. This study introduces a Do It Yourself (DIY) negative pressure suction (DIY-NPS) apparatus constructed with sterile gauze, hydrocolloid dressings, and disposable silicone gastrostomy tubes, ensuring therapeutic efficacy while lowering the treatment costs for pediatric patients’ families. Two pediatric DSWI cases treated with this device are presented in the following case report.

## 2 Case report

### 2.1 Patient 1

The first patient was a 1-month-old girl, born at 40 + 2 weeks of gestation, who developed shortness of breath and hypoxia for 24 days after birth. Diagnostic examinations revealed a ventricular septal defect, a patent ductus arteriosus, and tricuspid regurgitation. The patient underwent ventricular septal defect repair, patent ductus arteriosus ligation, and tricuspid valvuloplasty. The surgery lasted 5 h and 37 min, including 357 min on cardiopulmonary bypass, during which the chest was left open. Chest closure was performed on postoperative day 7. By postoperative day 12, the chest wound appeared cracked, red, and swollen, with purulent discharge. The wound measured 9 cm × 4 cm × 1.5 cm (Supplementary Figure S1), with a C-reactive protein (CRP) level of 99.21 mg/L and a white blood cell (WBC) count of 15.88 × 10^9^/L. Treatment included suture removal, debridement, application of the DIY-NPS for continuous suction (Supplementary Figure S12), intermittent saline irrigation, and maintenance of a negative pressure at −75 mmHg. The patient’s CRP level normalized by the third day of treatment. After 20 days of negative pressure therapy, the wound was clean, exhibiting fresh granulation tissue with no necrotic tissue or purulent discharge (Supplementary Figure S3). The wound was subsequently reclosed (Supplementary Figure S4). The sutures were removed on postoperative day 12, with complete wound healing. The patient was discharged after 59 days of treatment and demonstrated good wound healing during a follow-up 3 months post-surgery (Supplementary Figure S5).

### 2.2 Patient 2

The second patient was a 2-day-old boy, born at 37 + 5 weeks of gestation, who developed cyanosis 1 day after birth. Diagnostic examinations revealed complete transposition of the great arteries, a 5.2 mm ventricular septal defect, a minimal atrial septal defect, a patent ductus arteriosus, and moderate pulmonary hypertension. The patient underwent surgery to correct the transposition of the great arteries, close the ductus arteriosus, and repair the atrial septal defect. The surgery lasted 5 h and 22 min, including 220 min on cardiopulmonary bypass, during which the chest was left open. Chest closure was performed on postoperative day 4. By postoperative day 17, the wound appeared red and swollen, with purulent discharge and significant exudate. The wound measured 10 cm × 2.5 cm × 1 cm (Supplementary Figure S6), with a CRP level of 11 mg/L and a WBC count of 19.67 × 10^9^/L. Following debridement, the DIY-NPS (Supplementary Figure S7) was applied with continuous suction, intermittent saline irrigation, and a maintained negative pressure of −75 mmHg. The patient’s CRP level normalized by treatment day 2, and his WBC count normalized by treatment day 6. After 11 days of DIY-NPS therapy, the wound was clean, with well-formed granulation tissue and no significant necrotic tissue or abnormal exudate. The wound was reclosed (Supplementary Figure S8), the sutures were removed, and complete healing was achieved by postoperative day 13. The patient was discharged after 72 days of treatment.

## 3 Wound care

### 3.1 Formulating the wound care program

A bacterial culture from one child's wound revealed methicillin-resistant *Staphylococcus aureus* (MRSA), accompanied by significantly elevated CRP and WBC levels. These symptoms, including wound cracking, purulent discharge, oozing, redness, and swelling of surrounding tissues, met the criteria for an infected wound (7). Surgery, irrigation, drainage, and secondary closure remain the primary approaches for treating a DSWI. However, no standardized clinical strategy exists for sternal deep incision infections, leading to inconsistent outcomes. Furthermore, children exhibit a high failure rate and limited tolerance for invasive procedures. Internationally, Kadohama et al. first reported using closed continuous negative pressure suction (vacuum assisted closure (VAC)) to treat postoperative mediastinitis in children in 2007 (8). Taehee et al. (9), Renish et al. (10), and Lin et al. (11) also documented positive outcomes with VAC for managing deep incision infections in children post-heart surgery. The DIY-NPS aligns with the principles of VAC but offers advantages such as lower cost and simpler operation. DSWIs are characterized by full-thickness skin dehiscence, purulent discharge, high exudate, and an unstable sternum. Simple negative pressure suction facilitates effective drainage, promotes autolytic debridement, stabilizes the sternum, and stimulates granulation tissue formation for second-stage closure (10, 12).

### 3.2 Placement of a simple negative pressure device

#### 3.2.1 Preparation of the family and the child

A qualified nurse installed the negative pressure suction device after obtaining informed consent from the parents and explaining the procedure. Before placement, symptomatic management was implemented based on the child's pain assessment.

#### 3.2.2 Material preparation

1. Silver sulfate lipid hydrocolloid dressing,
2. Disposable 12F silicone gastric tube,
3. Medical surgical film,
4. Aseptic gauze,
5. Disposable sputum aspirator bottle, and
6. Center negative pressure device.

#### 3.2.3 Construction of the improvised device

Under strict aseptic conditions, the outer layer of a surgical film dressing was used to seal the wound surface. Silver-containing dressings filled the wound base, while a 12F silicone gastric tube and a silicone sputum suction tube served as the suction and flushing tubes, respectively. The size of the wound dictated the dimensions of the side holes in the tubing, which were cut at 1 cm intervals with a diameter of 2–3 mm. Negative pressure was set to −75 mmHg, and the suction and pressure device was replaced every 4–5 days.

### 3.3 Observation and care when using drainage devices

Continuous drip flushing was combined with constant negative pressure suction. Excessive rinsing may cause wound maceration and increase air leakage risk, while insufficient rinsing can hinder the removal of microorganisms, residues, and necrotic tissue, raising the likelihood of tube blockage. Intermittent rinsing has been shown to effectively control wound bacterial load (12). Thus, we observed the drainage fluid's color and characteristics during drip flushing, performed four times daily with 20 ml per session (10 ml every 5 min, followed by a 2-min pause). Negative pressure suction should be immediately discontinued if increased pain or active bleeding occurs (13).

## 4 Results

Two children with a DSWI received DIY-NPS therapy after precordial surgery, which was easy to implement and cost-effective. Both children were discharged successfully after 11–20 days of treatment.

## 5 Discussion

Guidelines for negative pressure therapy in infected chest wounds in children are lacking, and no uniform pressure values have been established. Studies by Obdeijn et al. (14), Vicchio et al. (15), and Renish et al. (10) report pressure ranges of −50 to −125 mmHg for pediatric chest incisions. The 2017 national expert consensus on using negative pressure closed drainage technology in burn surgery, formulated by several hospitals, recommended the following negative pressure values for children: −10.0 to −3.3 kPa (−75 to −25 mmHg) for children under 2 years, −10.0 to −6.6 kPa (−75 to −50 mmHg) for those aged 2–12 years, and −13.3 to −10.0 kPa (−100 to −75 mmHg) for adolescents aged 13–18 years (16). These values align with the pressures applied in this case. VAC includes a proprietary polyurethane foam dressing, connecting tubing, and a negative pressure pump (17), with costs ranging from hundreds to thousands of dollars. Postoperative care for children with congenital heart disease often requires prolonged hospitalization, imposing significant financial burdens on families. In contrast, the simple negative pressure device operates on the same principle but is cost-effective, easy to use, and highly practical. Silver ion dressings can inhibit biofilm formation, effectively control infection, and promote wound healing. Transparent dressings provide breathability and waterproofing while isolating the infected area, preventing cross-infection. These findings align with the studies by Zhan et al. and Wei et al. (18, 19). Despite promising results in applying closed drainage technology in pediatric cardiac surgery, several issues remain to be addressed. First, further research is needed to optimize the materials and design of negative pressure drainage devices for children, particularly regarding the appropriate negative pressure values for chest surgeries. Second, additional cases and data are required to assess the technique's efficacy and applicability across various pediatric cardiac surgeries.

## 6 Follow-up

The follow-up period confirmed the effectiveness and safety of the DIY-NPS device for managing pediatric DSWIs, with no observed long-term complications. These findings underscore the potential for broader application of the device in similar cases.

## Data Availability

The original contributions presented in the study are included in the article/Supplementary Material, further inquiries can be directed to the corresponding author.
