# Supplementary material for: Case Report: A review of two children with deep sternal wound infections after precordial surgery treated with a simple negative pressure closed drainage technique
Source: Front Pediatr. 2024 Dec 11;12:1491944. doi: 10.3389/fped.2024.1491944 (PMC11668580; doi:10.3389/fped.2024.1491944)
Supplement: Supplementary file 1 [file Image1.pdf]

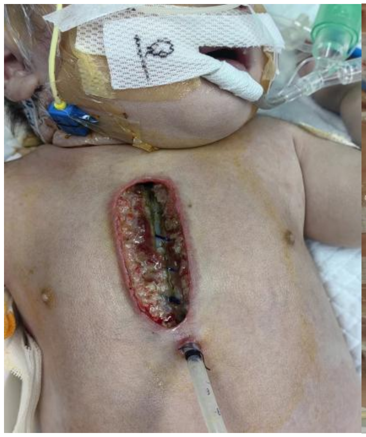

Fig.1

Postoperative wound on day 12

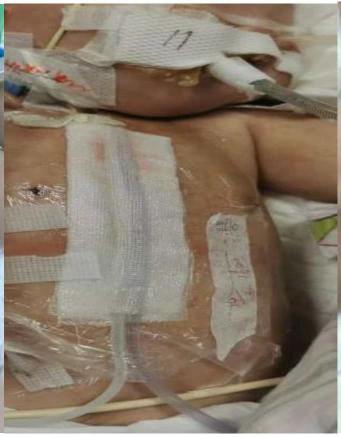

Fig.2

Simple negative pressure technique with continuous suction and intermittent saline irrigation

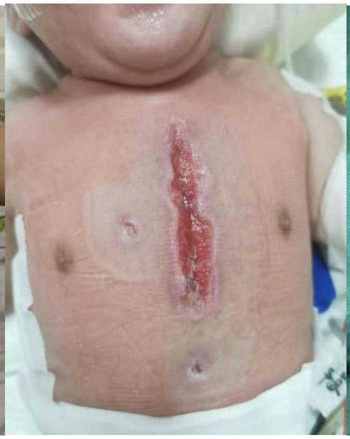

Fig.3

Wound cleaning after 20 days of negative pressure treatment

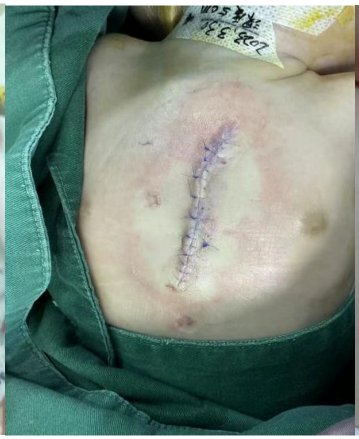

Fig.4

Secondary surgery for wound closure

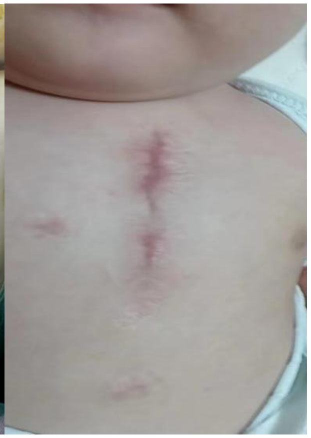

Fig.5

3-month postoperative follow-up

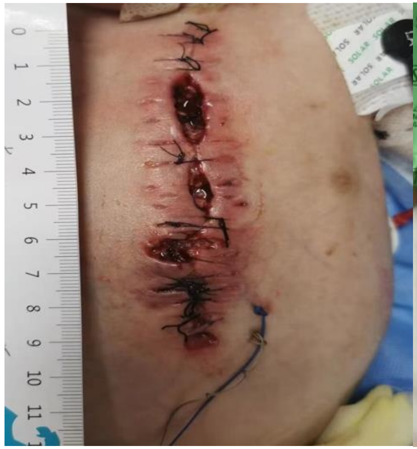

Fig.6

Postoperative wound on day 17

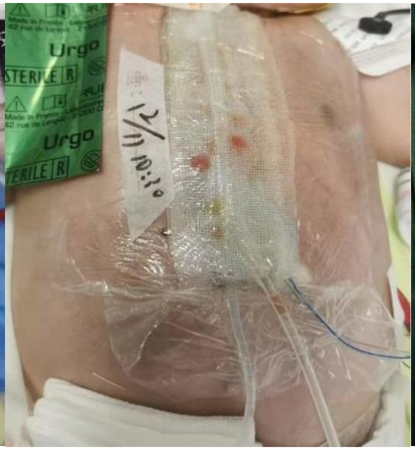

Fig.7

Simple negative pressure technique with continuous suction and intermittent saline irrigation

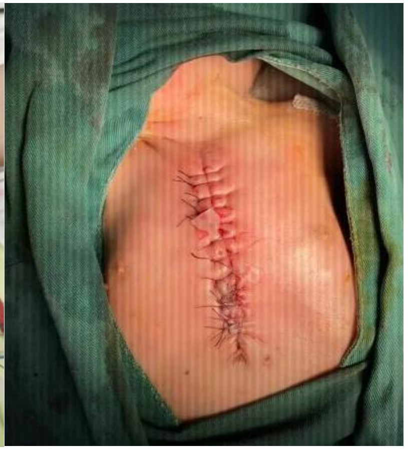

Fig.8

After 11 days of negative pressure treatment, a secondary wound
