# Supplementary material for: Case Report: A review of two children with deep sternal wound infections after precordial surgery treated with a simple negative pressure closed drainage technique
Source: Front Pediatr. 2024 Dec 11;12:1491944. doi: 10.3389/fped.2024.1491944 (PMC11668580; doi:10.3389/fped.2024.1491944)
Supplement: Supplementary file 2 [file Datasheet1.pdf]

Table

Case	Age (days)	Weight (kg)	Gender	Dignosis	Surgery	Delayed closure （days）	Wound condition	Pressure value (mmhg)	Negative pressure time （days）
1	30	2.5	girl	VSD<br>PDA<br>Tricuspid regurgitation	repair of ventricular septal defect,<br>ligation of patent ductus arteriosis，<br>tricuspid valvuloplasty	7	Purulent discharge, peritraumatic redness, high skin temperature, increased pain	-75	20
2	4	3.4	boy	TGA<br>PDA<br>ASD	transposition of the great arteries,<br>ductus arteriosus closure,<br>repair of atrial septal defect	4	Purulent discharge, heavy exudate, peritraumatic redness, high skin temperature	-75	11
